# Prevalence, incidence, and mortality of nontuberculous mycobacterial infection in Korea: a nationwide population-based study

**DOI:** 10.1186/s12890-019-0901-z

**Published:** 2019-08-01

**Authors:** Seon Cheol Park, Min Jin Kang, Chang Hoon Han, Sun Min Lee, Cheong Ju Kim, Jung Mo Lee, Young Ae Kang

**Affiliations:** 1 Division of Pulmonology, Department of Internal Medicine, National Health Insurance Service Ilsan Hospital, Goyang-si, Gyeonggi-do Republic of Korea; 2 Research Institute, National Health Insurance Service Ilsan Hospital, Goyang-si, Gyeonggi-do Republic of Korea; 3 Division of Pulmonology, Department of Internal Medicine, Yonsei University College of Medicine, 50-1 Yonseiro, Seodaemun-gu, Seoul, 03722 Republic of Korea

**Keywords:** Nontuberculous mycobacteria, Epidemiology, Prevalence, Incidence, Mortality

## Abstract

**Background:**

Epidemiologic characteristics of nontuberculous mycobacterial (NTM) disease remain largely unknown. The objective of this study was to evaluate incidence, prevalence, and mortality of NTM infection in a large nationwide population-based cohort in Korea.

**Methods:**

Data of the National Health Insurance Service database, an extensive health-related database including most Korean residents, were used. Adults with a primary diagnosis of NTM as determined by International Classification of Disease-Tenth Revision coding (A31) were identified between 2003 and 2016. Incidence, prevalence, and mortality of NTM infection were analyzed.

**Results:**

A total of 46,194 individuals had a primary diagnosis of NTM infection. Their mean age was 55.8 years. Of these subjects, 61.1% were females. Annual age-adjusted incidence and prevalence of NTM infection tended to increase rapidly from 2003 to 2016. Age-adjusted incidence and prevalence was 17.9 and 33.3 per 100,000 population in 2016. The incidence and prevalence were higher in females and the elderly. The 5-year mortality rate in the population with NTM infection was 17.8%. The standardized mortality ratio of patients with NTM infection to the general population was 2.16 (95% confidence interval: 2.10 to 2.22).

**Conclusions:**

This large population-based study showed that the incidence and prevalence of NTM infection in Korea increased rapidly from 2003 to 2016. They were higher in women and the elderly. The mortality rate in the population with NTM infection was higher than that in the general population.

## Background

Non-tuberculous mycobacteria (NTM) refer to mycobacterial species other than *Mycobacterium tuberculosis* complex or *M. leprae*. NTM are widely distributed in the environment. More than 160 species have been identified [1, 2]. The American Thoracic Society/Infectious Diseases Society of America (ATS/IDSA) guidelines recommended that the diagnosis of NTM pulmonary disease should be based on clinical, radiographic, and microbiologic findings [1]. The epidemiologic characteristics (especially incidence) of NTM pulmonary disease are difficult to estimate [3]. Therefore, many studies have used the epidemiology of NTM infection that is used when the standardized criteria were not met [3]. NTM infection was mainly confirmed by NTM isolation from a respiratory source in most studies.

The prevalence of NTM infection has increased worldwide in recent decades [4]. Improved diagnostic methods and increased physician awareness might have led to such increase in NTM infection. In addition, it has been suggested that its real prevalence is increased due to various causes such as changing demographics, with aging populations, increased comorbidities, and immunosuppression [5]. However, epidemiologic characteristics of NTM infection remain largely unclear. Unlikely tuberculosis, it is not mandatory to report NTM pulmonary disease to public health authorities in most countries [3]. Furthermore, data of previous epidemiologic studies are mainly from sentinel surveillance and microbiology laboratory-based results [3]. Results of studies on all-cause mortality of NTM pulmonary disease are also heterogeneous [5]. Long-term mortality results of NTM pulmonary disease or infection are mostly limited to single-center studies [6–9].

Korea has a unique single-insurer system that is the National Health Insurance Service (NHIS). It provides health insurance services to nearly all Korean residents and contains large-scale medical information. The purpose of this study was to estimate incidence and prevalence of NTM infection and the mortality in population with NTM infection using the NHIS database in Korea.

## Methods

### Data source and study population

Since 2000, all Korean citizens are obliged to register for the health insurance provided by the NHIS, and large amount of medical information could be accumulated in the NHIS database. The database in the NHIS system includes data on all healthcare use such as inpatient hospitalizations as well as outpatient visits to specialists or primary care physicians. In 2002, the NHIS built a population database containing personal information and medical treatment to provide useful data for health researchers. As of 2010, approximately 51 million Koreans were covered by NHIS system nationwide. Since national insurance in Korea is mandatory by law, the NHIS database contains data on all healthcare use of almost the entire population. We used this NHIS database to identify patients with NTM infection from 2002 to 2016.

### Diagnosis of NTM infection

Between 2002 and 2016, the NHIS database was used to identify individuals who visited at least one or more with a principal diagnosis of NTM infection coded as A31 according to the International Classification of Disease-Tenth Revision (ICD-10) coding system. For wash-out period, patients having NTM diagnostic codes in 2002 were excluded, and patients who were newly diagnosed with NTM infection between 2003 and 2016 were only analyzed.

### Demographics

Sex, age, region of residence, and Charlson comorbidity index (CCI) were analyzed between 2003 and 2016. The region of residence was categorized to metropolitan and other areas. CCI was calculated as previously described [10]. All variables were analyzed based on the time of NTM diagnosis.

### Outcomes

The prevalence of NTM infection was defined as the number of individuals visiting medical institutions who were assigned ICD-10 code A31 for NTM as the principal diagnosis. Only patients who visited the medical institution that year were included in the prevalence analysis because we tried to exclude patients who no longer suffer from NTM infection without using the medical institution. For incidence analysis, we used three different criteria. First, a new case of NTM infection was defined at a patient’s first visit with ICD-10 code A31 assigned as the principal diagnosis. Second, a new case of NTM infection was defined at a patient’s first visit with A31 code as the principal or additional diagnosis. Third, a new case was defined at a first visit in a patient having at least two or more visits with A31 code as principal or additional diagnosis. We used the first inclusion criterion as the primary method of incidence analysis and compared it with the other two inclusion criteria. If a person was identified as NTM multiple times, only the first identification was included in the incidence analysis.

For mortality analysis, individuals were followed up until the end of the study period or death. The time of death was recorded in year and month. The standardized mortality ratio was used to compare the mortality rate of those with NTM infection to that of the general Korean population. The immediate cause of death in the death certificate was used to classify the cause of death, including tuberculosis (A15–19), pneumonia (J12–18), chronic lower respiratory disease (J40–47), lung cancer (C34), other cancer (C00–97, except C34), cardiovascular disease (I20–25, I30–52), cerebrovascular disease (I60–69), diabetes mellitus (E10–14), and hypertension (I10–15) according to the Korean Standard Classification of Disease and Cause of Death-Sixth revision.

### Statistical analysis

Descriptive statistics were performed for all variables. Annual NTM-related visits by sex, age, and region of residence were analyzed for incidence and prevalence. The general population living in Korea was used as the denominator for the prevalence and incidence analysis. The population during the middle of 2010 was used as a standard statistical population. Kaplan-Meier analyses were performed to calculate the overall survival of NTM patients by sex, age, and CCI. The SAS software, version 9.4 (SAS Institute, Cary, NC, USA) was used for all statistical analyses.

## Results

### Baseline characteristics

A total of 46,194 patients with NTM were identified between 2003 and 2016 (Table 1). Their mean age was 55.8 ± 19.5. Females accounted for 61.1%. Most patients were diagnosed with NTM infection over 50 years of age. Of all patients, 45.7% were living in metropolitan areas and 96.0% of had more than one point for CCI.

**Table 1:** Baseline characteristics of patients with NTM infection, 2003~2016

	Total
Total	46,194
Male	17,979 (38.9)
Female	28,215 (61.1)
Mean age	55.8 ± 19.5
0–19	1,836 (4.0)
20–29	2,895 (6.3)
30–39	4,192 (9.0)
40–49	5,886 (12.7)
50–59	9,489 (20.5)
60–69	9,596 (20.8)
70–79	8,998 (19.5)
≥80	3,302 (7.1)
Region of residence	
Metropolitan	21,121 (45.7)
Others	25,073 (54.3)
Charlson comorbidity index	
0	1,841 (4.0)
1	5,639 (12.2)
2	11,224 (24.3)
3	6,442 (13.9)
4	6,107 (13.2)
5	14,941 (32.3)

Values are presented as number (%) or mean ± standard deviation

*NTM* nontuberculous mycobacteria

### Prevalence

**Fig. 1:**
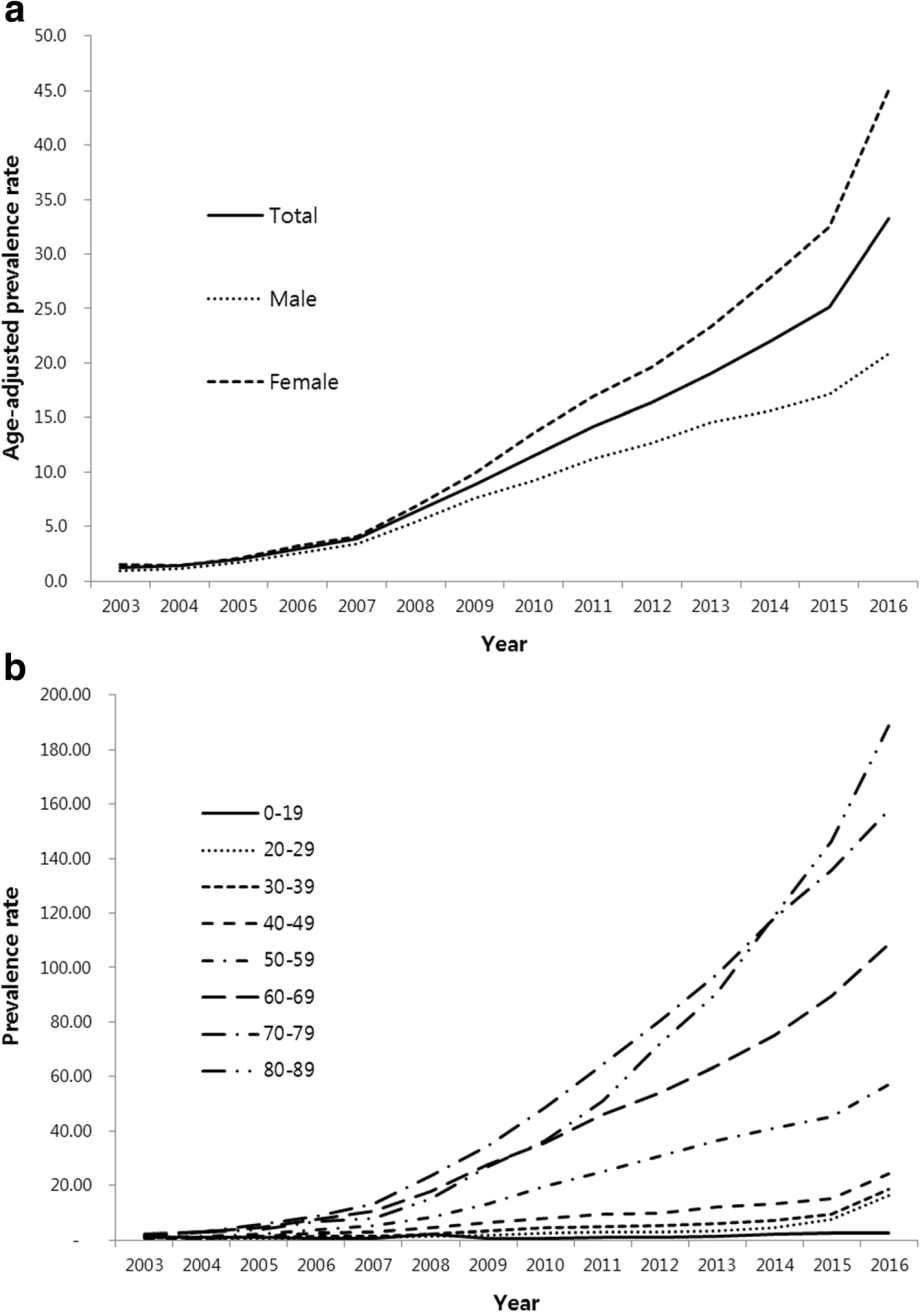
Prevalence of NTM infection between 2003 and 2016 (**a**) and by age groups (**b**). The rates are per 100,000 population. NTM = nontuberculous mycobacteria

Age-adjusted prevalence of NTM infection rapidly increased from 2003 to 2016. The age-adjusted prevalence was 1.2 per 100,000 population in 2003 and 33.3 per 100,000 population in 2016 (Fig. 1). The age-adjusted prevalence of NTM infection was higher in women. In 2016, it was 20.8 per 100,000 population in men and 45.1 per 100,000 population in women. The prevalence of NTM infection was also increased with age. In 2016, the prevalence was the highest in patients aged 80 years or older (188.7 per 100,000 population).

### Incidence

**Fig. 2:**
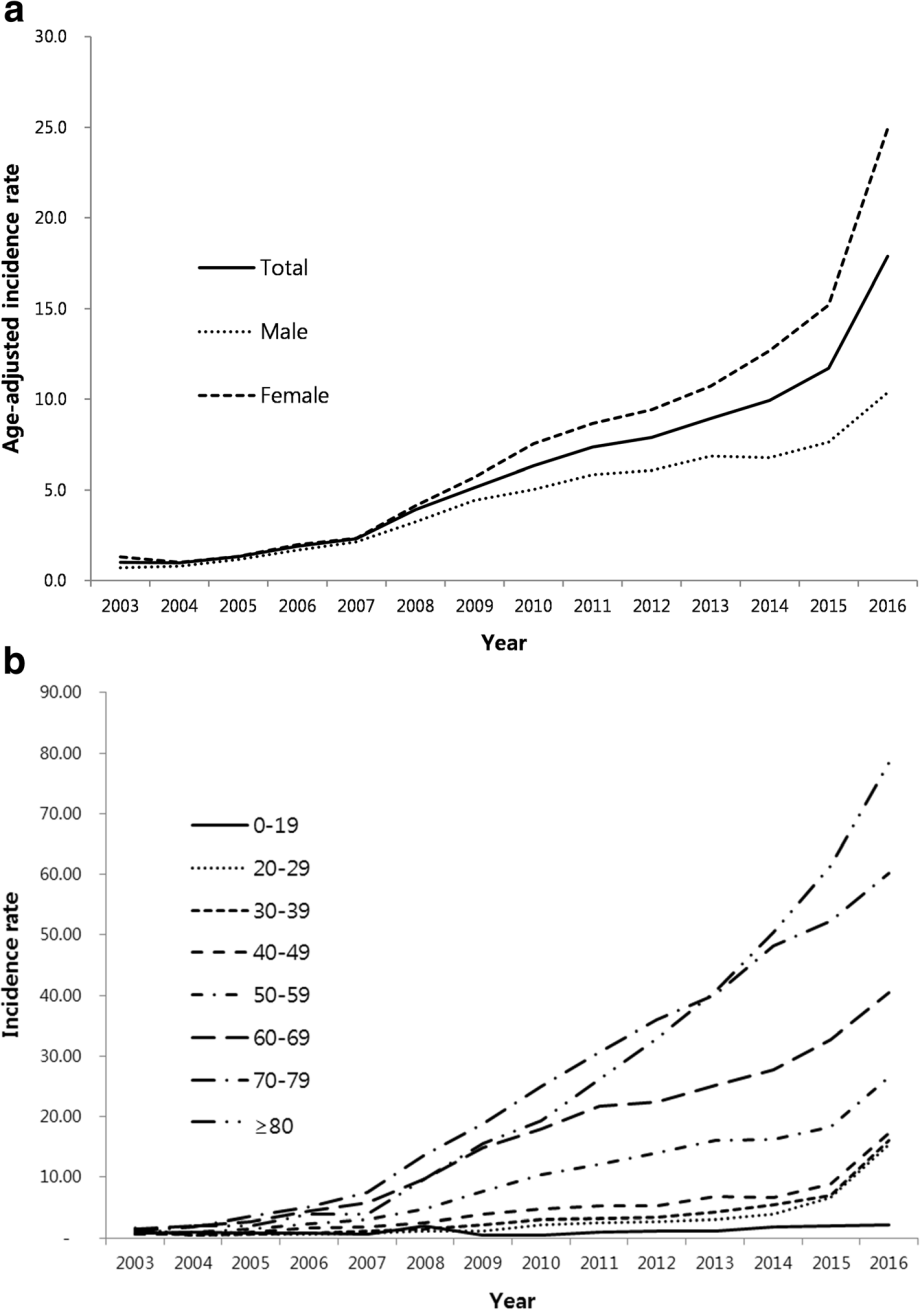
Incidence of NTM infection between 2003 and 2016 (**a**) and by age groups (**b**). The rates are per 100,000 population. NTM = nontuberculous mycobacteria

Similar to prevalence, age-adjusted incidence of NTM infection was also increased from 2003 to 2016 (Fig. 2). The age-adjusted incidence was 1.0 per 100,000 population in 2003 and 17.9 per 100,000 population in 2016. Women had about 2.5 times higher age-adjusted incidence than men (24.9 vs. 10.4 per 100,000 population). Elderly patients also had higher incidence.

**Fig. 3:**
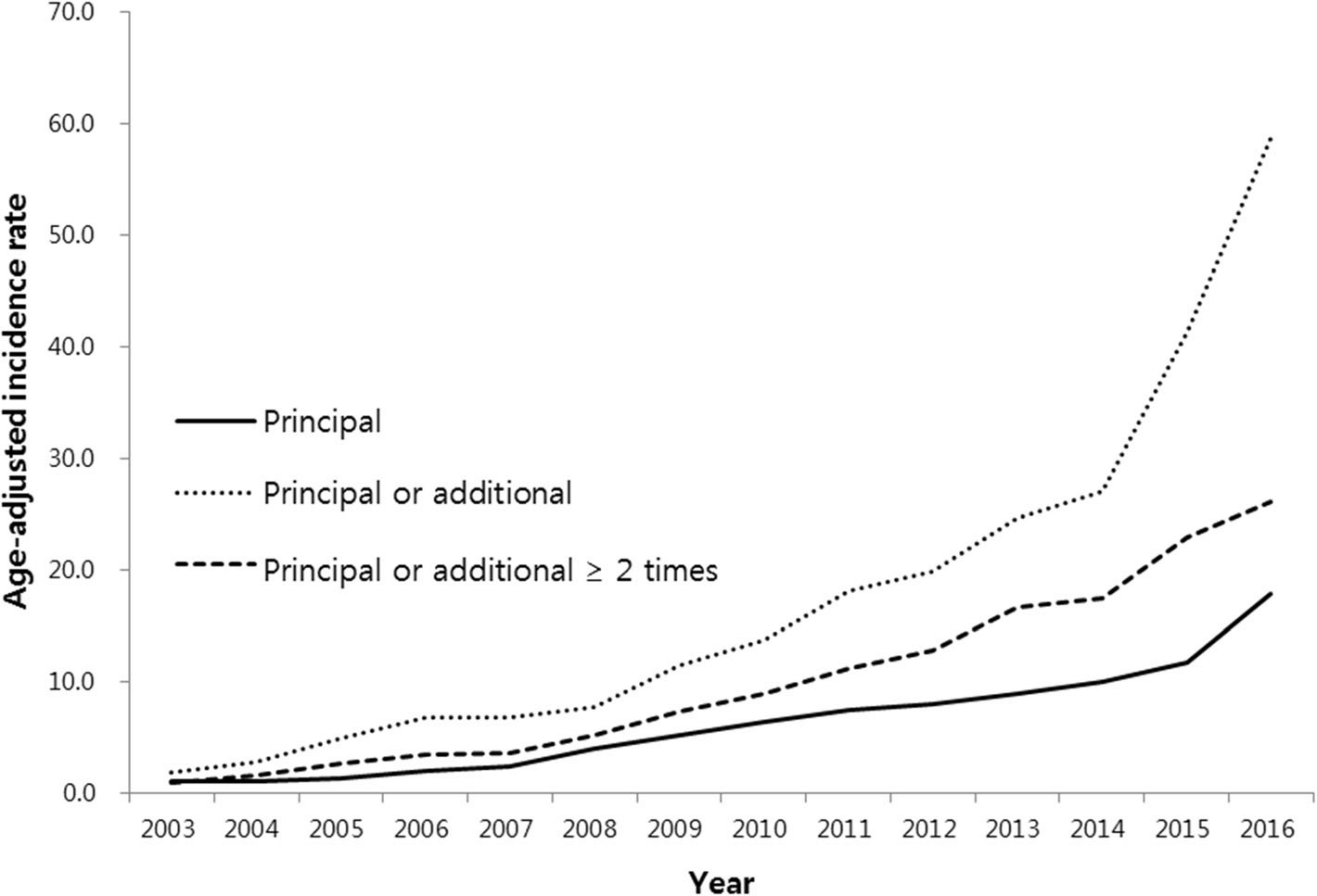
Incidence of NTM infection between 2003 and 2016 by different diagnostic criteria. The rates are per 100,000 population. Principal: One or more visits with NTM diagnostic code as principal diagnosis; Principal or additional: One or more visits with NTM diagnostic code as the principal or additional diagnosis; Principal or additional ≥2 times: Two or more visits with NTM diagnostic code as the principal or additional diagnosis. NTM = nontuberculous mycobacteria

According to different diagnostic criteria, there were differences in the incidence of NTM infection (Fig. 3). The total number of patients was 46,194 using the inclusion criteria of one or more visits with A31 code as principal diagnosis. It was 128,710 using the inclusion criteria of one or more visits with A31 code as principal or additional diagnosis and 74,144 when using two or more visits with A31 code as principal or additional diagnosis. In each of these three groups of patients, the age-adjusted incidence of NTM infection in 2016 was 17.9, 58.7, and 26.1 per 100,000 population, respectively.

### Mortality

**Fig. 4:**
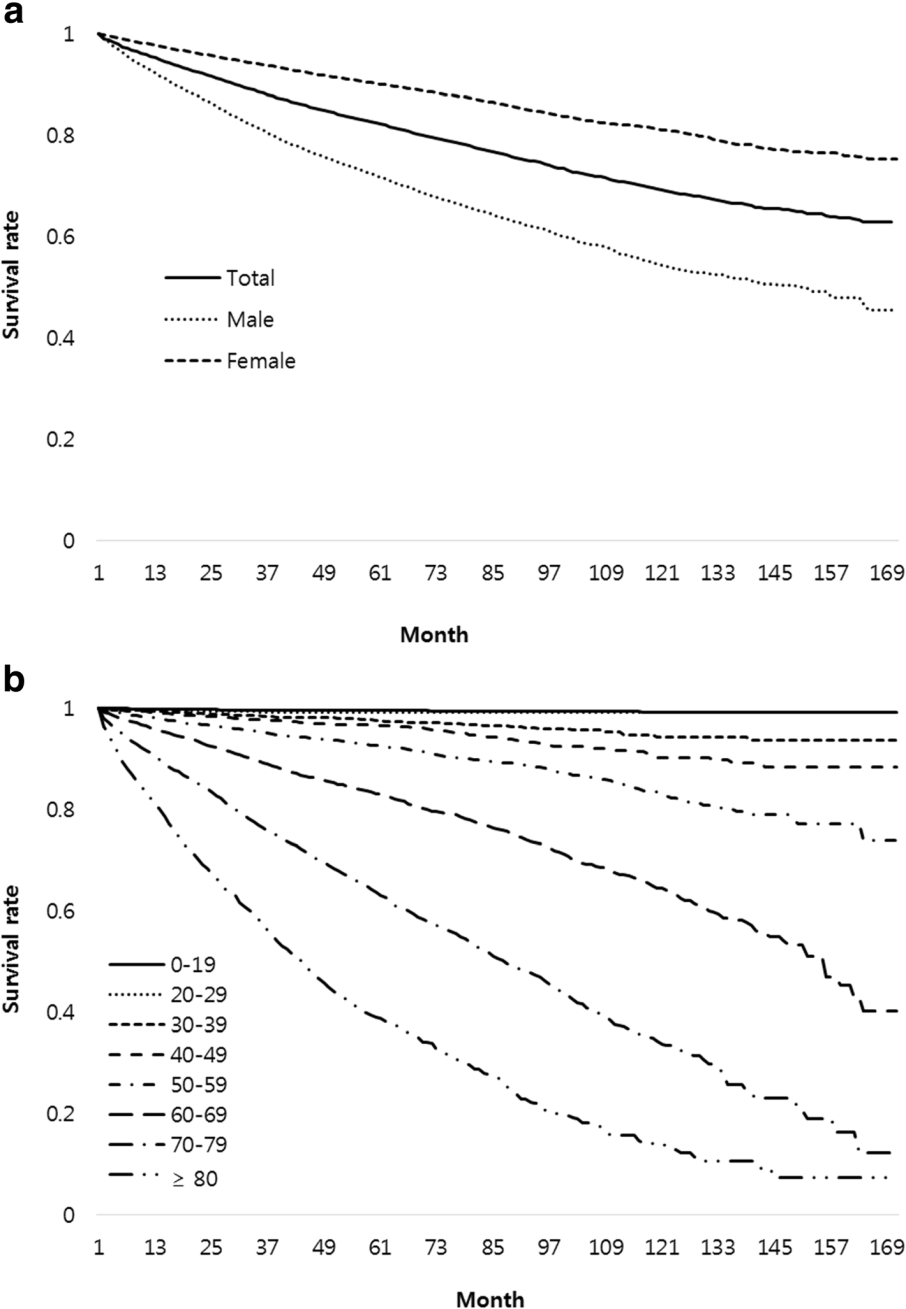
Survival curve of patients with NTM infection by gender (**a**) and by age (**b**). NTM = nontuberculous mycobacteria

There were a total of 5,112 deaths in patients with NTM infection during the study period. Mean follow-up period was 41.8 ± 36.5 months. Mortality rates of all patients with NTM infection at 1 year and 5 years after diagnosis were 4.7 and 17.8%, respectively (Fig. 4). The 5-year mortality rate was about three times higher in men than that in women (28.3% vs 9.9%). The mortality rate increased with age and CCI. The standardized mortality ratio of patients with NTM infection to the general population was 2.16 (95% confidence interval [CI]: 2.10 to 2.22). Common causes of death in patients with NTM infection were respiratory diseases such as tuberculosis (10.0%), pneumonia (8.3%), and chronic lower respiratory disease (14.2%), and lung cancer (7.2%). Other cancers (15.0%) were also common causes of death (Table 2).

**Table 2:** Cause of death in patients with NTM infection

Causes of death	Number of patients (%)
Tuberculosis	509 (10.0)
Pneumonia	424 (6.0)
CLRD	725 (8.3)
Lung cancer	369 (14.2)
Other cancers	769 (7.2)
CDVD	300 (15.0)
CBVD	155 (5.9)
DM	99 (3.0)
Hypertension	34 (1.9)
Others	1,392 (27.2)
Unknown	29 (0.6)
Total	5,112 (100.0)

*CLRD* chronic lower respiratory disease, *CDVD* cardiovascular disease, *CBVD* cerebrovascular disease, *DM* diabetes mellitus

## Discussion

To the best of our knowledge, this is the largest population-based study of NTM epidemiology. The age-adjusted prevalence and incidence rapidly increased from 2003 to 2016 in Korea. Both prevalence and incidence were higher in women and the elderly. The mortality rate of patients with NTM infection was higher than that of the general population and the most common cause of death was respiratory disease.

It is difficult to determine the exact epidemiologic features of NTM pulmonary disease or infection due to differences in study methodologies and underlying populations. The epidemiology also varies depending on regional differences and the time of investigation. However, most studies have clearly shown that the prevalence and incidence of NTM pulmonary disease or infection tend to increase worldwide, including Korea [11–18].

Our measures of incidence and prevalence of NTM infection may have underestimated the actual prevalence and incidence. In our study, the prevalence and incidence of NTM infection rapidly increased from 2003 to 2016. The prevalence increased about 28 times and the incidence increased 18 times during the study period. This pattern might not be a real underlying increase. NTM infection might have been underestimated in the past two decades in Korea. Enhanced physician awareness and improved detection methods could explain the observed increase of NTM infection [19]. For example, the utilization of computed tomography has increased continuously during our study period in Korea [20]. The universal healthcare coverage system in Korea has improved the access to medical institutions and increased mycobacterial cultures which might also contribute to the increased detection of NTM infection. For this reason, we suspect that our methods are not accurately to yield represent true incidence and prevalence pattern during the study period. Under-diagnosis or under-utilization of the diagnostic codes may have led to falsely low estimates early in the study period, and perhaps the figures toward the end of the study period are closer to accurate at the population level. This underscores the critical importance of repeating these analyses further into the future.

The epidemiology of NTM pulmonary disease or infections has remained largely unclear in Korea. However, recent NTM epidemiology studies have shown that NTM pulmonary disease or infections are increasing steadily [17, 21, 22]. One study conducted in two Korean tertiary-care hospitals reported that the incidence of NTM pulmonary disease increased from 6.8 per 100,000 patients in 2009 to 12.9 per 100,000 patients in 2015 [21]. Another study conducted in a tertiary referral hospital similarly reported that the incidence of NTM pulmonary disease increased from 7.0 per 100,000 patients in 2001 to 55.6 per 100,000 patients in 2015 [22]. As in our study, another single center study has determined the prevalence of NTM infection using coding data from the Health Insurance Review and Assessment Service (HIRA) in Korea and reported that the prevalence of NTM infection per 100,000 population is increased from 9.4 in 2009 to 36.1 in 2016 [17]. This increasing trend of prevalence was similar to our study because the HIRA database shared health-related data with the NHIS database used in our study.

Globally, the prevalence and incidence of NTM pulmonary disease or infection are also increased [19]. A recent study performed in five states in the United States has reported that age-adjusted prevalence of NTM infection is increased from 8.7 per 100,000 population in 2008 to 13.9 per 100,000 population in 2013 [14]. Similarly, another study from United States-affiliated Pacific Islands has reported that the prevalence of patients with NTM infection is steadily increased from 2 cases to 48 cases per 100,000 persons during 2007–2011 [15]. A study from Germany has also shown that the prevalence rate of NTM pulmonary disease is increased from 2.3 to 3.3 cases per 100,000 population during 2009–2014 [18]. A multicenter study from Nagasaki in Japan has reported that the incidence of NTM pulmonary disease increased from 4.1 to 10.1 per 100,000 population during 2001–2009 [12].

Our study showed that the prevalence and incidence of NTM infection were increased with age. Most previous studies that examined NTM pulmonary disease or infection have also reported similar results [12, 14, 18, 23–27]. Considering the increase in aging population worldwide, the high prevalence and incidence of NTM infection in the elderly are particularly important because increased NTM infection may increase socioeconomic burden of NTM pulmonary disease. In some countries including Korea, an increase in NTM pulmonary disease or infection has occurred simultaneously with a decrease in tuberculosis which is another important infectious disease [13, 16, 21, 23].

Our study showed that the overall ratio of female to male patients with NTM infection was 1.57 and the incidence of NTM infection in 2016 was about 2.5 times higher in women than that in men. However, gender differences in the prevalence and incidence of NTM pulmonary disease or infection were heterogeneous among studies. Some studies have reported that there are no differences in the prevalence or incidence of NTM pulmonary disease or infection between men and women while other studies have reported higher prevalence and incidence in women [15, 18, 23–25, 27].

NTM pulmonary disease requires long-term treatment [1]. Long-term prognosis of NTM pulmonary disease is not well-known. Population-based studies for mortality are lacking. Several population-based studies have evaluated the long-term mortality of NTM pulmonary disease or infection. A population-based study from Ontario has shown that both NTM pulmonary disease and NTM pulmonary isolation without disease are associated with higher rates of death compared to propensity score-matched control [28]. The hazard ratio (HR) for death was 1.47 (95% CI: 1.42 to 1.51) compared to control. The 5-year mortality was 26.6% for NTM pulmonary isolation and 36.9% for NTM pulmonary disease. A study using Oregon population-based cohort has reported that patients with NTM respiratory isolates have high mortality (5-year mortality of 35.1%) regardless of whether they meet the ATS/IDSA criteria for NTM pulmonary disease [29]. Another population-based study from Denmark has observed that the 5-year mortality in patients with definite NTM pulmonary disease is 40.1% which is slightly higher than that in patients with NTM colonization (33.5%) [30]. The 5-year mortality of patients with NTM infection in our study was lower than that in previous studies (17.8% vs. 26.6 to 35.1%). Our study included relatively young patients which might explain such difference. The mean age of patients with NTM infection at diagnosis was 56 years in our study while the mean or median age in previous studies was 60 to 70 years. Similar to Ontario study, our results showed that patients with NTM infection had higher mortality compared to the general population.

Our study has limitations. First, we defined NTM infection using only ICD-10 code assigned by health care providers. Our coding-based analysis could not examine the laboratory results, patient records, or radiologic results. We relied on physician judgment for diagnosis of NTM infection. Although we analyzed the epidemiology of NTM infection using a variety of working definitions, we could not know exactly what results would be most similar to the real NTM epidemiology in Korea. Unfortunately, there is no study to confirm the accuracy of the coding definition of NTM in Korea. Diagnosis of NTM pulmonary disease is difficult for several reasons, including variable symptoms of patients, need for computed tomography, presence of the organism in the environment, a disease definition based on scant evidence, and no obligation to report the disease [3, 31]. Most epidemiologic studies for NTM pulmonary disease have suffered these challenges. Second, the prevalence of NTM infection might be underestimated compared to its incidence. NTM infection may often remain life-long in patients because of its chronic feature of inflammation and frequent re-infection. Therefore, the annual prevalence can increase in proportion to its cumulative incidence. For analysis of annual prevalence, we only included patients visiting medical institutions that year. Therefore, patients who did not visit medical institutions regularly might have been excluded from the prevalence analysis. Third, the specific sites of NTM infection could not be analyzed. Our analysis was limited using the less specific A31 code because more specific codes (A31.0 and A31.1) were infrequently specified in the NHIS databases.

## Conclusions

This study showed that the prevalence and incidence of NTM infection in Korea, as measured by coding data, rapidly increased in the recent two decades. The prevalence and incidence were higher in women and the elderly. Considering such increase in the aging population, the prevalence and incidence may increase further in the future. The mortality in those with NTM infection was approximately twice than that in the general population. This trend should be closely monitored to establish optimal healthcare policies and treatment strategies for NTM infection.

## Data Availability

The data that support the findings of this study are available from National Health Insurance Sharing Service in Korea, but restrictions apply to the availability of these data, which were used under license for the current study, and so are not publicly available.

## Abbreviations

ATS/IDSA: American Thoracic Society/Infectious Diseases Society of America
CCI: Charlson comorbidity index
CI: Confidence interval
HIRA: Health Insurance Review and Assessment Service
HR: Hazard ratio
ICD-10: International Classification of Disease-Tenth Revision
NHIS: National Health Insurance Service
NTM: Nontuberculous mycobacteria
US: United States
